# Single-stage total hip arthroplasty after failed fixation of proximal femoral fractures: an increased risk for periprosthetic joint infections?

**DOI:** 10.1007/s00402-021-04119-0

**Published:** 2021-08-28

**Authors:** P. Hemmann, F. Schmidutz, M. D. Ahrend, S. G. Yan, U. Stöckle, A. J. Schreiner

**Affiliations:** 1 Department of Traumatology and Reconstructive Surgery, BG Trauma Center Tübingen, Eberhard Karls University Tübingen, Schnarrenbergstrasse 95, 72076 Tübingen, Germany; 2 Department of Orthopaedic Surgery, Physical Medicine and Rehabilitation, University of Munich (LMU), Munich, Germany; 3 Department of Orthopaedic Surgery, The First Affiliated Hospital of Anhui Medical University, Hefei, China; 4 Center for Musculoskeletal Surgery (CMSC), Charité University Hospital Berlin, Berlin, Germany

**Keywords:** Infection, PJI, Femoral fracture, Diagnostics

## Abstract

**Background:**

Higher complication rates have been reported for total hip arthroplasty (THA) after osteosynthesis of proximal femur fractures (PFF). This study evaluated the infection risk for conversion of internal fixation of PFF to THA by a single-staged procedure in the absence of clear infection signs.

**Methods:**

Patients undergoing a one-staged conversion to THA (2013–2018) after prior internal fixation of the proximal femur were included. Preoperative diagnostics with laboratory results, hip aspirations as well as intraoperative microbiology and sonication were assessed. Postoperative complications were recorded as well as patient demographics, duration between initial and conversion to THA, explanted osteosynthesis and implanted THA.

**Results:**

Fifty-eight patients (24 male/34 female, 62.8 ± 14.5 years) were included with a mean time of 3.8 ± 7.5 years between internal fixation and conversion to THA (45 cementless, 3 cemented, 3 hybrid and 7 hybrid inverse THAs). Preoperative mean blood level CRP was 8.36 ± 14 mg/l (reference value < 5 mg/l) and leukocyte count was 7.11 ± 1.84^3/µl (4.5–10.000^3/µl). Fifty patients had intraoperative microbiological diagnostics, with either swabs in 86.2% and/or sonication in 29.3%. Positive microbiological results were recorded in 10% (5 of 50 patients), with pathogens identified being mainly Staphylococcus. Complications after conversion occurred in 9.6% including a postoperative low-grade infection rate of 5.8% after a mean of 2.5 years.

**Conclusion:**

This study found a positive microbiological test result in 10% of a one-stage conversion of PFF fixation to THA. Moreover, we found a high infection rate (5.8%) for early postoperative periprosthetic joint infection. Interestingly, CRP has not been proven to be an adequate parameter for low-grade infections or occult colonized implants. Therefore, we recommend a comprehensive pre- and intraoperative diagnostic including hip aspiration, swabs and sonication when considering one-staged revision.

## Introduction

Proximal femoral fractures are one of the most common fractures with an incidence between 150 and 250/100,000 each year in industrial nations [1]. These fractures predominantly occur in elderly aged of more than 65 years (90%) [2] and the incidence will further increase due to epidemiological development [3–5] with an expected doubling over the next 25 years [6].

Treatment usually includes internal fixation e.g., dynamic hip screw or intramedullary nails to preserve the natural hip joint. Nevertheless, high failure rates of up to 45% have been reported [6–9] and are related to femoral head necrosis, pseudarthrosis, cutting-out, peri-implant fractures as well as infection [10–12].

Revision usually results in conversion to total hip arthroplasty (THA). Unfortunately, studies reported clearly higher complication rates compared to primary THA [13, 14], including higher rates of dislocations, loosening and periprosthetic joint infection (PJI) [15, 16].

Periprosthetic infections are a devastating complication in elderly patients and might be referred to the multimorbid patient collective but also to previous surgery with orthopedic implants inserted. These implants are prone to occult infections [17] and are a result of bacteria adhesion and biofilm formation at the implant surface. Up to 50% occult infection rates of orthopedic implants have been reported [18] and can preoperatively not reliably be excluded with clinical and laboratory analysis, resulting in an increased risk for PJI [17, 19, 20].

Up to now, only few studies, most with small collectives, reported the outcome following surgical revision to THA. Thus, there is currently no international consensus, whether conversion of failed fixation to THA should be performed as a one- or two-stage procedure to reduce the risk of PJI. A single-stage procedure offers the advantage of only one surgical procedure and rapid mobilization on these elderly patients, while it potentially increases the risk of PJI.

The aim of this study was to evaluate the risk of PJI in one-staged conversion from failed fixation of PFF to THA in the absence of clinical infection signs.

## Materials and methods

The hospital database was retrospectively (June 2013 to June 2018) screened for patients undergoing a one-staged conversion to THA after prior osteosynthesis of the proximal femur.

Patients were included with a history of a proximal femoral fracture (femoral neck fracture, trochanteric fracture) with initial osteosynthesis (dynamic hip screw, screws, intramedullary nail, plate) following a one-staged conversion to THA (cemented, cementless, hybrid, inverse hybrid THA).

Exclusion criteria were defined as no implantation of THA due to diagnosed or obvious signs of peri-implant or soft tissue infection.

The following parameters were retrospectively collected based on: gender, age at conversion, date of initial surgery, date of conversion surgery, duration between those two surgeries, preoperative blood sample levels of CRP and leukocytes, explanted osteosynthesis material (screws, dynamic hips screw, intramedullary nail, screws, plate) and the type of implanted THA (cemented, cementless, hybrid, inverse hybrid THA). Moreover, if available, results of preoperative diagnostic hip aspirations (alpha-defensin, leukocytes, percentage of polymorphic cells) as well as intraoperative microbiology and sonication were collected and evaluated. However, preoperative diagnostic hip aspiration and intraoperative sonication were not used routinely in our hospital at that time.

The records were further screened for postoperative complications defined as soft tissue infection, PJI or revision surgery after THA. The follow-up was performed on our out-patient clinic.

Patient demographics and clinical parameters are presented descriptively as mean ± standard deviation (minimum–maximum).

Receiver-operating characteristic (ROC) was calculated to analyze the test performance of preoperative CRP levels as a predictor for positive intraoperative swabs or PJI. We determined *p* ≤ 0.05 as significant. Statistical analysis was performed with Microsoft© Office Excel 2016 (Microsoft Corporation, Redmond, USA) and with JMP® (SAS Institute Inc., version 14.2, Cary, NC, USA). The study protocol was approved by the local ethics committee (803/2018BO2) of the university.

## Results

### Patient collective

After screening the records, a total of 58 patients (24 men, 34 women) were eligible for inclusion. The mean age at conversion surgery was 62.8 ± 14.5 years (19.0–91.3). The mean time between initial surgery and revision was 3.8 ± 7.5 years (0–30.6). Early revision (≤ 6 months) was necessary in 15 patients (26%). The type of internal fixation removed included 17 screws, 26 dynamic hip screws, 7 intramedullary nails and 8 plates. Conversion to THA included 45 cementless THAs, 3 cemented THAs, 3 hybrid and 7 hybrid inverse THAs (Table 1), whereas revision components were used in 8 cases (13.8%). 4 patients received revision stems, while 1 patient required a Burch–Schneider reinforcement cage and 1 patient needed a trabecular metal cup-cage construct. 2 patients received revisions stems and tripolar cups.

**Table 1:** Demographic and clinical data (mean ± standard deviation (minimum–maximum) or n (%)) of the cohort

Demographics of the cohort (*n* = 58)	
Age at revision	62.8 ± 14.5 years (19.0–91.3)
Gender	24 (41%) male; 34 (59%) female
Data regarding surgeries	
Duration first surgery and THA implantation	3.8 ± 7.5 years (0–30.6)
Explanted osteosynthesis	17 (29%) screws
	26 (45%) dynamic hip screws
	7 (12%) intramedullary nails
	8 (14%) plates
Implanted THA	45 (78%) cementless THA
	3 (5%) cemented THA
	3 (5) hybrid THA
	7 (12%) inverse hybrid THA
Data regarding infection and its diagnostic	
Preoperative CRP	8.36 ± 14 mg/l (0.1–87)
Preoperative leucocytes count	7.11 ± 1.84^3/µl (3.3–11.2)
Number of available intraoperative microbiological swabs	50 (86.2% of the total cohort)
Number of available intraoperative sonication	17 (29.3% of the total cohort)
Intraoperative positive cultures	5 (10% of available swabs, 1 swab pos. with 2 pathogens)
Intraoperative detected pathogens	1 *S. aureus*
	3 *S. epidermidis*
	1 *S. warneri*
	1 *S. saccharolyticus*
Follow-up cohort (*n* = 52) and observed complications	5 (9.6%) overall complication rate
	3 (5.8%) infection
	1 (1.9%) aseptic cup loosening
	1 (1.9%) aseptic stem loosening

### Preoperative laboratory

The preoperative laboratory examination showed a mean CRP value of 8.36 ± 14 mg/l (0.1–87) (reference value < 5 mg/l) and a mean leukocyte count of 7.11 ± 1.84^3/µl (3.3–11.2) (reference value 4.5–10.000^3/µl). 23 patients presented an elevated CRP and 5 patients an elevated leukocyte count.

### Intraoperative microbiology and sonication

Microbiological swabs were available in 86.2% (50 of 58 patients) and implant sonication in 29.3% (17 of 58 patients), with 29.3% (17 of 58 patients) having microbiology and sonication. Overall, a positive culture was found in five patients (10%).

In two patients, the microbiological swabs were positive whereby sonication was negative in one case and not performed in the other. Pathogens identified were *S. aureus* (*n* = 1) and *S. epidermidis* (*n* = 1).

In three patients, the microbiological swabs and sonication were positive. Identified pathogens were *S. warneri* (*n* = 1), *S. epidermidis* (*n* = 2) and *S. saccharolyticus* (*n* = 1), with one patient showing two pathogens (*S. saccharolyticus*, *S. epidermidis*).

### Preoperatively diagnostic hip aspiration

In 14 cases (24.1%), a preoperatively diagnostic hip aspiration was performed with one case being a sicca aspiration.

From the aspiration, microbiological cultures were examined in 92.8% (13 of 14 patients) with detection of *S. epidermidis* in one culture.

Alpha-defensin was also evaluated in these patients 92.8% (13 of 14 patients) but did not reveal a positive result: 0.1–0.2 (> 1.1 high risk for infection).

Leukocyte cell count and percentage of polymorph cells could be analyzed in 57% (8 of 14 patients), with 5 patients showing a cell count < 1000/µl, 1 patient 1000–3000/µl and 2 patients > 3000/µl. The polymorph cells showed a range of 19–75% with a mean percentage of 49.5%

### Postoperative therapy

All patients received an antibiotic prophylaxis intraoperatively, after taking the microbiological swabs. Antibiotic therapy was postoperatively continued until the final results were available after 14 days. In case of positive microbiological examination, patients received 6 weeks of combination therapy including two antibiotics to which the bacteria were susceptible.

### Follow-up

During the follow-up, two patients died who were not associated with conversion surgery. Another three patients were lost to follow-up due to lack of presentation. One patient moved abroad. The mean follow-up time of the remaining patients (*n* = 52) was 2.5 years (0.5–5.6). Complications occurred in 9.6% of the patients, with one aseptic cup loosening (1.9%), one aseptic stem loosening (1.9%) and three low-grade infections (5.8%). From these patients, none of them had a positive pre- or intraoperative microbiological result.

**Fig. 1:**
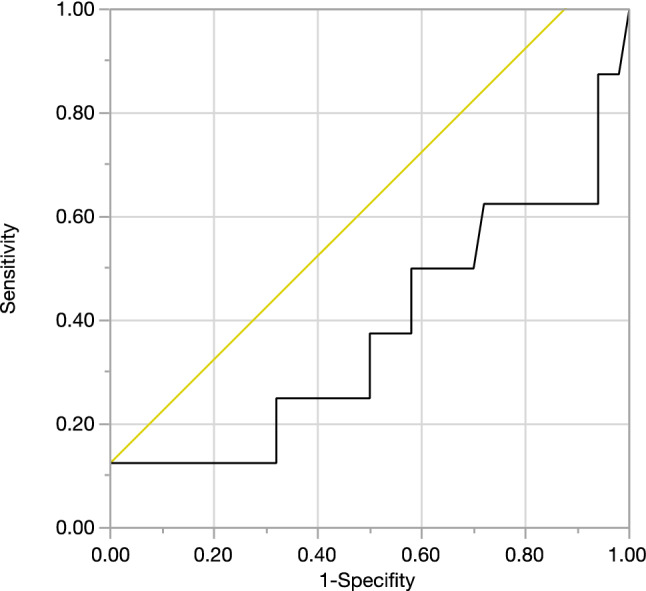
Receiver-operating characteristics (ROC) curve for preoperative CRP as predictor for intraoperative positive microbiological swab or PJI. CRP was not a good predictor for an intraoperatively positive swab or later infection

**Fig. 2:**
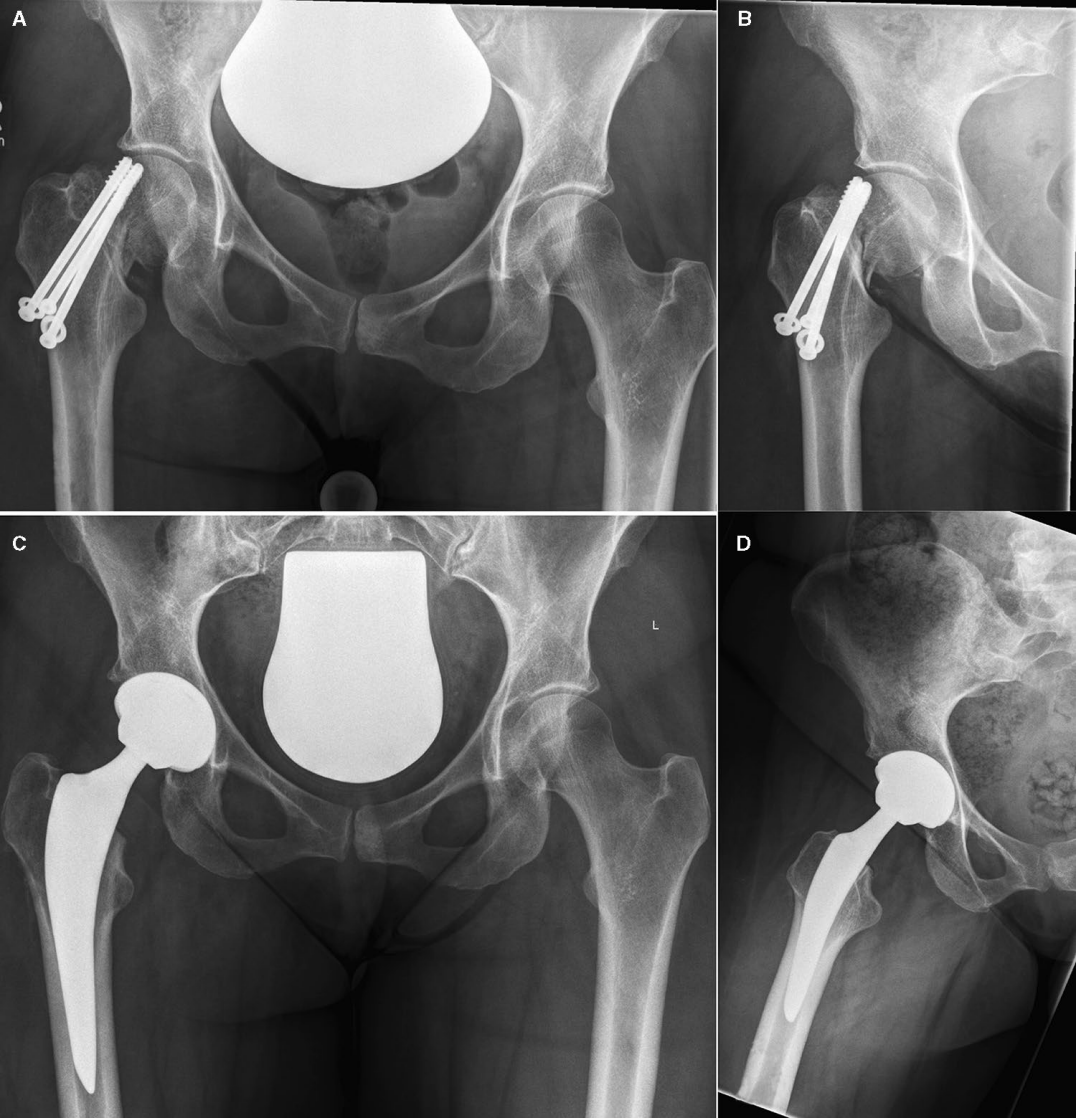
49-year-old woman with failed screw osteosynthesis right 9 months after femoral neck fracture. THA 6 weeks after conversion surgery

In eight patients, neither microbiological swabs nor sonication was available. One of the three low-grade infections was diagnosed within this group during follow-up. All other complications which were listed above were detected in the microbiologically tested group. Receiver-operating characteristics (ROC) curve for preoperative CRP as predictor for intraoperative positive microbiological swab or PJI was not significant (*p* = 0.3793).CRP (AUC: 0.3775) demonstrated to be a poor fit (Fig. 1). An example of a patient requiring conversion surgery from internal fixation to THA is given in Fig. 2.

## Discussion

This study evaluated whether a revision from internal fixation of proximal femoral fractures to THA can safely be performed as a one-staged procedure. Our results show a relevant number of intraoperative positive microbiological swabs and also higher revision rate compared to primary THA.

Positive microbiological results were found in 10% of the patients, despite the preoperative lack of clinical infection signs. Comparable to our results, Gittings et al. evaluated the conversion in 33 patients from internal femoral fixation to THA and found a high infection rate of 18% [21]. Kempthorne et al. found a rate of 15% positive cultures in patients with aseptic loosening for explanted THA or TKA implant (*n* = 54) [19]. These rates of occult infection are similarly reported in several studies evaluation removal of inconspicuous orthopedic implants [22, 23]. Knabl et al. evaluated osteosynthesis implants during routine removal after long bone fracture. The removed implants were assessed by sonication and PCR and the authors reported a positive microbiological culture in 56% [24]. Another study by George et al. showed a positive culture rate of 51% in the explanted implants before THA [25]. In accordance to our and other studies, all patients had no clinical signs for infection prior implant removal [24]. The lower rate of positive results in our study compared to other studies [21, 24] is probably related to the different evaluation methods used, with PCR and sonication showing higher detection rates. Taking only our sonication results into account (3 out of 17), the positive rate also increases to 17.6% (*n* = 3) in our collective.

The importance of sonification is strengthened by results of aseptic revisions in joint arthroplasty. Trampuz et al. documented a rate of 9% positive sonication cultures in a series of 54 removed hip and knee implants [26]. Rothenberg et al. reported for assumed aseptic revisions in THA and TKA a positive sonication rate of 15% [27]. Some studies certify sonication a higher sensitivity for microbiological diagnostic than the conventional methods [17, 28], but the higher rate of false-positive results has also to be noted [29, 30]. Nevertheless, sonication seems to be an essential part to improve detection and treatment of occult infection [28].

In our study, Staphylococcus (*S. epidermidis*, *n* = 3), *S. aureus*, *S. saccharolyticus* and *S. warneri* (*n* = 1) were the predominant microorganism detected which is in accordance with previous studies describing coagulase-negative Staphylococcus as the most prevalent organism on osteosynthesis and arthroplasty devices [19, 20, 24, 27]. Coagulase-negative Staphylococcus are well known to produce a protective layer of biofilm [20] which does not induce acute inflammatory response, but results in low-grade infections [23, 26]. Sonication is able to destroy the protective biofilm and allows the detection of those bacteria in the microbiological culture [23]. As a consequence, all patients with an intraoperative positive microbiological swab in our study were treated successfully with a calculated double antibiotic therapy for at least 6 weeks, according to an early PJI of THA.

The high colonization rate of osteosynthesis devices in clinical healthy patients is nowadays well known and a trigger for PJI [24]. However, it is not always clear whether implant failure (e.g., loosening) is caused by low-grade infection or infection is supported in the environment of loosening [19]. Still in all patients undergoing conversation to THA, an occult infection caused by a primary internal fixation must be considered [31] although detection is still challenging [32].

A preoperative laboratory with white cell blood count (WBC) and ESR or CRP are recommended but are nonspecific markers for PJI for low-grade infections in arthroplasty [33]. Gittings et al. showed that these markers are useful tools although occult infection could be missed [21]. They propose that patients with borderline or elevated inflammatory markers should be highly suspicious for infection. In our cohort, four out of five patients who had intraoperative positive microbiological swabs showed non elevated CRP blood levels postoperatively and 1 patient demonstrated a slightly elevated CRP value. In our cohort, the CRP blood level was not a good predictor for detecting occult colonized implants or low-grade PJI as seen by the ROC curve performed in this study. Pérez-Pietro et al. could also show that blood inflammatory markers like CRP level, or ESR may not be accurate enough especially when identifying low-grade and chronic PJI [34]. Therefore, aspiration with several tests such as, cell count, alpha-Defensin and microbiology seems to be a helpful tool for prevention of a PJI [35, 36]. If preoperative a PJI is suspected, we nowadays prefer a two-staged revision, although other authors still recommend a one-staged revision with antibiotics [37, 38] in favor of a single operation and a faster mobilization.

A complication rate of 9.6% was recorded during our postoperative follow-up. This rate appears to be high for THA, especially considering an early PJI rate of 5.8%. Nevertheless, the data are in line with the current literature, and several studies even reported clearly higher numbers with a PJI risk ranging from 1.3 to 18% [13, 21, 39–41]. Several reasons might influence the increased infection risk and include the prolonged operation time [42], scar tissue in the approach for hip exposure, synovitis and hip stiffness and higher risk for intraoperative fractures due to osteopenia [16]. However, the PJI seems to be the highest risk due to the occult infection of the osteosynthesis devices. All patients diagnosed with a positive microbiology result were successfully treated with a combination therapy of two antibiotics for 6 weeks.

As a consequence of this retrospective study documenting the number of detected occult infections, we adopted our pre- and perioperative regime: all patients now receive: (1) preoperative laboratory incl. CRP and white cell count; (2) preoperative aspiration including a-Defensin, cell count with polymorph cells, CRP aspiration, Leucocyte Esterase test and microbiology/culture from the aspirate and (3) perioperative microbiological swabs/probes and sonication of the removed implants should follow to diagnose PJI. If preoperatively an (occult) infection is suspected according to the latest criteria of Parvizi et al. [43], a two-stage revision is performed. If one of the perioperative cultures is positive, we continue the combination therapy including two antibiotics for 6 weeks.

This study has several limitations which were connected to the retrospective study design. First, the study only analyzed patients with a single-stage revision and no comparative group with two-stage revision or primary THA was available. Second, the mean follow-up was relatively short and the evaluated data were presented descriptively in a heterogenous cohort. Third, the increasing knowledge in diagnostic PJI has clearly changed the concept of preoperative diagnostics which will clearly alter the outcome. Fourth, the postoperative infection rate might be underestimated since only 52 patients were evaluated for follow-up which implies a probable higher complication and infection rate than described. Nevertheless, it should be mentioned that nearly all complications occurred within the microbiologically tested group (which can, therefore, be considered a reliable testing tool).

## Conclusion

Overall, we intraoperatively found an increased rate of positive microbiological results (10%) during a one-staged revision when converting internal fixation of proximal femur fractures. We also found an increased risk for a PJI (5.8%) which is in line with the current literature. However, in contrast to the increase PJI risk, the one-staged revision offers a faster mobilization and only one surgical procedure, potentially reducing peri- and postoperative complications. Interestingly, CRP has not been proven to be an adequate parameter for low-grade infections or occult colonized implants. In consequence of the results of this study, we recommend extensive pre- and intraoperative diagnostics including hip aspiration, microbiological swabs and sonication and in case of preoperative pathogen detection consideration of a two-staged procedure.
